# Dysplasia of the fibrous sheath with axonemal and centriolar defects combined with lack of mitochondrial activity as associated factors of ICSI failure in primary ciliary dyskinesia syndrome

**DOI:** 10.1590/S1677-5538.IBJU.2019.0362

**Published:** 2020-12-20

**Authors:** Juliana R. Pariz, Caroline Rané, Joel Drevet, Jorge Hallak

**Affiliations:** 1 Laboratório de Andrologia Clínica e de Pesquisa de Alta Complexidade Centro de Ciência e Inovação em Andrologia São PauloSP Brasil Androscience - Centro de Ciência e Inovação em Andrologia e Laboratório de Andrologia Clínica e de Pesquisa de Alta Complexidade, São Paulo, SP, Brasil; 2 Universidade de São Paulo Faculdade de Medicina Divisão de Urologia SP Brasil Divisão de Urologia, Faculdade de Medicina da Universidade de São Paulo, SP, Brasil; 3 Universidade de São Paulo Faculdade de Medicina Departamento de Patologia SP Brasil Unidade de Toxicologia Reprodutiva - Departamento de Patologia, Faculdade de Medicina da Universidade de São Paulo, SP, Brasil; 4 Université Clermont Auvergne Faculty of Medicine GReD Institute Clerrmont-Ferrand France GReD Institute, Faculty of Medicine, CRBC building, Université Clermont Auvergne, Clerrmont-Ferrand, France; 5 Universidade de São Paulo Instituto de Estudos Avançados (IEA-USP) SP Brasil Instituto de Estudos Avançados (IEA-USP), Universidade de São Paulo, SP, Brasil

## INTRODUCTION

Primary ciliary dyskinesia (PCD) is a rare autosomal recessive disorder characterized by ciliary dysfunction and impaired mucociliary clearance, resulting in a range of clinical manifestations, including chronic bronchitis leading to bronchiectasis, chronic rhino-sinusitis and otitis media, situs inversus (in about 50% of cases) and severe male infertility (1). PCD was initially characterized in men with Kartagener syndrome in 1982 by ultrastructural analysis that demonstrated dyskinetic or immotile cilia, but not limited to this particular diagnosis (2).

The incidence of PCD is estimated at 1/16.000 births based on the prevalence of situs inversus and bronchiectasis (3). In addition, the prevalence of PCD may be much higher in some communities and countries, particularly when inbreeding is present (4). Severe asthenozoospermia and up to complete immobility of the sperm are the main known causes of male infertility in patients with PCD, due to the inability of sperm to reach and fertilize the oocyte correctly. However, if this was the only reason, one would expect that with the use of assisted reproduction techniques (ARTs), particularly when the sperm is injected directly inside the oocyte, therefore without the physiological evolutionary ability to bypass the oocyte plasma membrane and cumulus cells, such as the invasive resourceful intracytoplasmic sperm injection (ICSI), in which one sperm is injected directly into the oocyte, the inability found in the PCD patient to conceive would be more commonly bypassed in the vast majority of cases. Surprisingly, exactly on the contrary, there are only a handful number of reports of successful pregnanies that resulted in childbirth among couples in which the male partner is affected by PCD syndrome and its variants (5). Even when the most advanced ARTs have been used, including alleged intracytoplasmic injection of morphologically selected sperm (IMSI), time lapse monitoring of IVF-produced embryos, preimplantation genetic diagnosis and screening, a high percentage of PCD male partners never get pregnant. The involuntary childlessness critically found in this group of individuals, suggests that in addition to the incapacity of sperm to reach the oocyte, there might be other unknown factors acting. This prompted us to use the arsenal of tests available in a high-complexity andrology laboratory focused in the understanding of sperm physiology, biochemistry and ultrastructure to have a thorough evaluation of the functional and structural characteristics of sperm in PCD syndrome after unsuccessful attempts of IVF/ICSI and establish a helpful guidance for similar situations.

### ICSI and Primary ciliary dyskinesia syndrome

**Figure 1:**
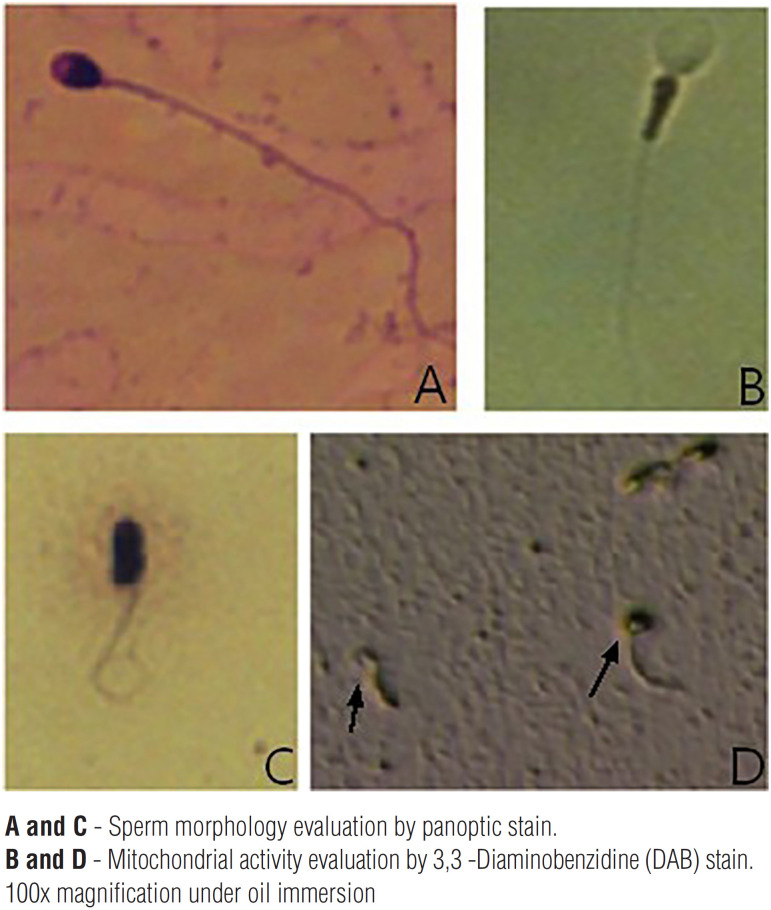
Sperm morphology and mitochondrial activity representative photographs. A) Normal human sperm morphology according to WHO criteria is defined as follows: sperm head should be smooth, regularly contoured and generally oval in shape, a well-defined acrosomal region comprising 40-70% of the head area should be seen, the midpiece should be slender, regular and about the same length as the sperm head, the major axis of the midpiece should be aligned with the major axis of the sperm head. Residual cytoplasm is considered an anomaly only when in excess, the principal piece should have a uniform caliber along its length (45µm long, approximately 10 times the head legth) and be thinner than the midpiece. Image courtesy of the Androscience. B) Normal sperm mitochondrial activity representative photograph with DAB dye accumulation in the sperm midpiece. Image courtesy of Androscience Lab. C) Example of coiled tail and amorphous spermatozoa in PCD spermatozoa. D) Representative photograph of defective DAB staining observed in PCD spermatozoa. Arrows demonstrate absence of mitochondrial activity by DAB stain and amorphous spermatozoa can be visualized.

**Figure 2:**
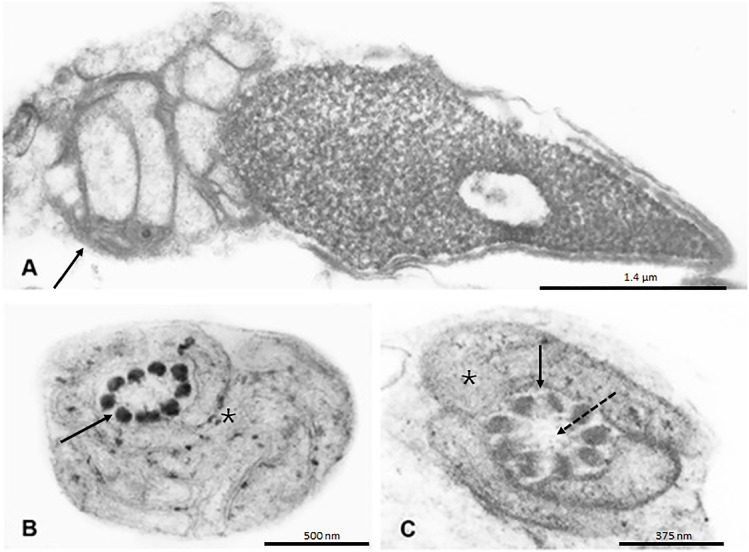
A) Ultrastructural longitudinal section showing cross section of the sperm head and midpiece. Chromatin presents a granular appearance with hypodense lacunar defects. The mitochondrial sheath (left arrow) is seen as a conglomerate of ultra-structurally disorganized and overlapping mitochondria. Centriole cannot be properly found and correctly located. B) Transverse section of the sperm midpiece showing the initial part of the flagellum with its 9 outer dense fibers (arrow) and disorganized mitochondria sheath do not form a helically arranged single row (*). C) Transverse section of midpiece showing flagellum (arrow). The microtubular doublets of the axoneme are not clearly seen and the central pair is missing (dashed arrow). Disorganized mitochondria sheath does not form a helically arranged single row (*).

**Figure 3:**
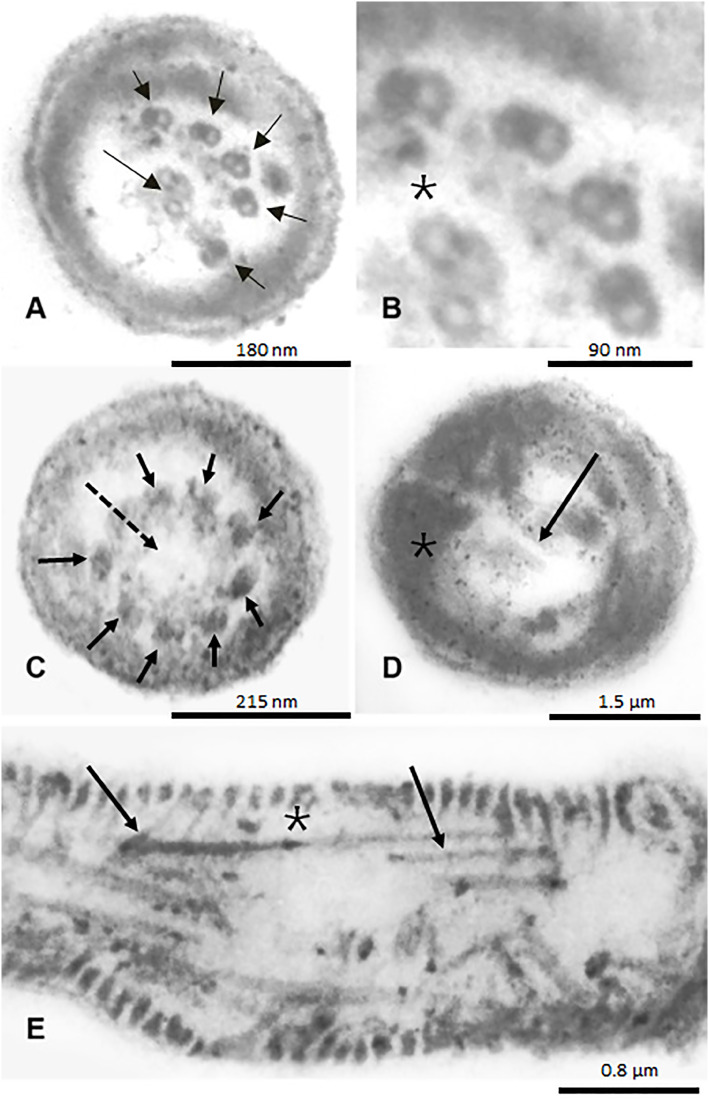
Ultrastructural images representing sperm flagella at the distal end of the principal piece. A and B) Four microtubular doublets of the axoneme are missing. The central pair and the peripheral doublets 1 to 5 are clearly visible (arrows). Note the absence of nexin links, outer and inner dynein arms in all doublets (*). C) Another sperm flagella. The central pair is missing (dashed arrow) and axonemal configuration is 9 + 0 (small arrows). D) Absence of doublets of axoneme and central pair (arrow). The fibrous sheath is thickened, disorganized and duplicated (*). E) Longitudinal section of the principal piece showing disorganized hyperplastic fibers of the fibrous sheath (arrows) and dense external longitudinal fibers (*).

**Figure 4:**
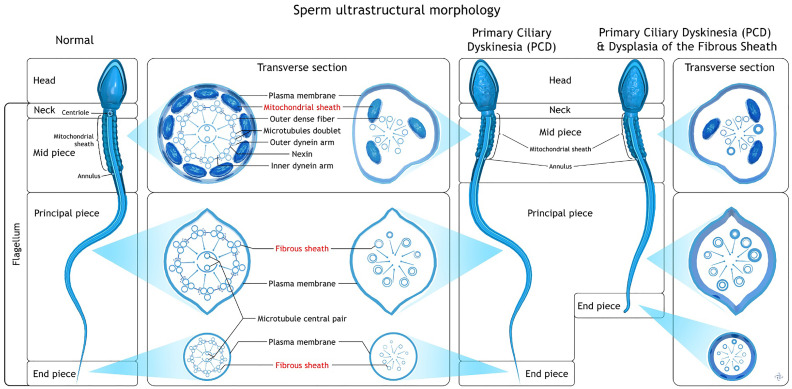
Comparative ultrastructural morphology of normal spermatozoa, primary ciliary dyskinesia (PCD) and PCD with dysplasia of the fibrous sheath. Illustration developed by Androscience.

A 26-year-old male (66kg, 179cm; BMI=21.53 kg/m^2^), asthmatic, with primary infertility and a self-reported situs inversus, along with his partner, a healthy 22-year-old woman with eumenorrhea, was referred for an andrological evaluation after failure in IVF attempts. The couple was first evaluated by a reproductive gynecologist who requested a single conventional semen analysis (6) that revealed total immotility, and proceeded directly to IVF/ICSI, which unfortunately is a common direct next-step decision in many of male infertility cases without proper workout (7). Complete lack of fertilization was the result of two IVF/ICSI attempts and before starting a third new cycle of ovulation induction, the couple decided to move for a second opinion with an Andrologist (JH). Physical examination revealed normal sized testicles, presence of bilateral vas deferens and normal epididymis as well as the absence of varicocele or any other scrotal abnormalities. Seminal parameters were then further investigated and data are presented in Table-1. Sperm vitality assessed by Hoechst-33258 fluorescent staining demonstrated a mean of 86% viable sperm (8). Sperm morphology revealed 100% amorphous sperm with a high proportion of tails rolled up or coiled (Figure-1C, normal morphology is illustrated in Figure-1A, for comparison). Round cell counts and qualitative evaluation of fructose were normal. As recommended in cases of severe asthenozoospermia, we performed sperm cultures after prostate massage for aerobic and anaerobic bacteria, including *Mycoplasma hominis, Ureaplasma urealyticum* and *Chlamydia trachomatis*, and no bacterial growth was observed (9). To assess the redox status of the seminal sample, we quantified the presence of reactive oxygen species (ROS) by chemiluminescence (10) and sperm mebrane lipid-peroxidation levels by detection of reactive thiobarbituric acid substances (TBARS) (11, 12). Both were considered within normal parameters. Immaturity of the sperm cells was assessed by measuring creatine kinase (CK) activity (13) and again, no increase in the percentage of immature spermatozoa was identified (Table-1). Mitochondrial activity was assessed using a technique based on the oxidation of 3,3′-Diaminobenzidine (DAB) with cytochrome C oxidase. In this test, the reagent is polymerized and deposited on the mitochondrial sheath along with the sperm midpiece. In that assay, the accumulation of dye in the middle piece is considered to be proportional to the mitochondrial activity of the sperm (14). We found no stains in the median part of our patient's semen, indicating a severe decrease, almost absence in mitochondrial activity (Figure-1D, normal stain illustrated in Figure-1B, for comparison). Sperm DNA fragmentation was monitored using the sperm chromatin structure assay (SCSA®) and flow cytometry (15). We recorded a moderately/high index of sperm DNA fragmentation (% DFI >30%, see Table-1) that could help to explain reproductive failures. Electron microscopy (EM) analysis was finally added as a valuable investigation resource to further clarify sperm cell typical or non-typical flagellar and morphological defects associated or not with this pathology. In summary, EM analysis of one hundred spermatozoa revealed short, very thick and irregular tails, with fibrous sheath abnormalities, not found in all cases of immotile cilia. Therefore, these alterations were present in 100% of evaluated spermatozoa. Typically, the normal 9+2 axonemal structure was deformed and many microtubular profiles were completely obliterated, with total absence of dynein arms, nexin links and possible other ultrastructure defects not easily observed even in electron microscopy, and only described by a highly experienced expert in sperm EM. Center pairs were also missing (9+0 configurations) in most (but not all) flagella. The fibrous sheath was strongly enlarged, revealing a serious disturbance of the flagellar ultrastructure, likely to have a direct causal link with total fertilization failure presented here. The mitochondria, normally organized in a regular sleeve of simple elements, arranged in a helix manner, were much fewer in number and grouped in various layers superimposed around the axoneme and dense outer fibers, with serious lack of spermatozoa function. This resulted in complete disorganization of the neck and central part of the spermatozoa. The sperm centriole was displaced with a disturbed internal ultrastructure. Small alterations in the organization and compaction of sperm chromatin have been observed in the form of nuclear gaps and vacuoles in 38% of spermatozoa. All these abnormal spermatozoa characteristics are illustrated in Figures 2, 3 and 4.

**Table 1:** Seminal analysis performed in accordance to The World Health Organization (WHO) 2010 guidelines, patient results and reference values.

Parameter	Patient results		Reference value
	Sample #1	Sample #2	
pH	8.0	8.0	>7.2
Semen volume	5.0mL	6.0mL	≥1.5mL
Sperm concentration	33 million/mL	35 million/mL	15 million/mL
Total sperm number	165 millions	210 million	≥39 million
Progressive motility	0%	0%	≥32% progressive sperm
Total motility	0%	0%	≥40% motile sperm
Vitality	88% live spermatozoa	84% live spermatozoa	≥58% live spermatozoa
Sperm morphology	0% normal forms	0% normal forms	≥4% normal forms
Round cells	0.75 millions/mL	2.0 millions/mL	<1 million/mL
Seminal fructose	Positive	NA	Positive
Reactive oxygen species (ROS)	0.14×10^4^cpm/20×10^4^ spermatozoa	NA	0.55×10^4^ cpm/20×10^4^ spermatozoa (10)
Lipid peroxidation (LPO)	182.69mg x 10^5^ of TBARS/10^6^ spermatozoa	NA	*Range 130.7 - 201.7mg x 10^5^ of TBARS/10^6^ sperm is suggested as low lipid peroxidation (12)
Creatine kinase activity (CK)	0.05U/10^8^ spermatozoa	0.03 U/10^8^ spermatozoa	*Range 0.09 - 0.15 IU/10^8^ spermatozoa in fertile volunteers with normozoospermia (13)
DNA fragmentation index (DFI)	NA	33% fragmentation	DFI <25% (22)
Sperm mitochondrial activity (DAB)	Non-stained, <5%	Non-stained, <5%	*(14)

* No reference values established yet.

Athayde et al., 2007 (10); Camargo et al., 2014 (12); Hallak et al., 2001 (13); Evenson, 2016 (22); Hrudka, 1987 (14).

## DISCUSSION

In this study, we present an in-depth andrological evaluation of spermatozoa and seminal parameters in a patient with PCD syndrome with severe dysplasia of the fibrous sheath, in order to better understand the unexpected results of total fertilization failure in two IVF/ICSI attempts in a very young couple. As expected in the context of PCD and in accordance with early descriptions of classical PCD sperm phenotypes under electron microscopy (1, 16), we observed that our patient had axonemal sperm alterations, lack of dynein arms and pairs of central microtubules, associated with centriole absence. These alterations were also accompanied by severe flagellar defects, including short, thick and curled sperm tails, as well as a complete disorganized midpiece compartment, lack of most part of the mitochondrial sheath. The fibrous sheath was disorganized, hypertrophied with serious disruption of flagellar ultrastructure.

The modified phenotype of the sperm mitochondria was confirmed as no DAB staining could be observed in the spermatozoa of this PCD patient further evidencing a highly defective mitochondrial functional system. Extreme mitochondrial system dysfunction in PCD syndrome may help in the understanding of the low to very-low fertilization results in men for whom advanced assisted reproductive techniques were attempted such as IMSI. Even when the micromanipulation lab is technically capable of performing highly advanced sperm selection techniques after laborious sperm processing together with incubation procedures with motility-enhancement substances such as caffeine or oxidative-stress protectors such as melatonin (17, 18). It is therefore assumed that the abnormal axonemal structures and a defective mitochondrial energy supply system could explain the immotility of PCD sperm and their inability to fertilize naturally.

In addition, the lack of centriole may also partly explain the low success rate of ICSI with PCD sperm, since it has been demonstrated that the sperm centriole is fully responsible for the nucleation of microtubules and formation of a functional mitotic spindle in early human embryos, therefore being a key element for the opposition and alignment of both male and female embryo pronuclei to properly oppose and constitute the embryonal genome in early stages of embryo development (19, 20).

Another critical observation came from the fact that creatine kinase levels were within normal range, reflecting that in the PCD phenotype, sperm abnormalities are likely to be limited to the midpiece and flagellar structures without significantly affecting neither the biochemical nor the cytological maturation/differentiation stages intrinsic to the phase of spermiogenesis that reshapes the spermatozoa head. The sperm head with and the normal Sertoli-assisted resorption of the cytoplasmic droplet in the spermiogenesis that might otherwise reveal immaturity, energy synthesis failure and functional inefficiency of the spermatozoa (13).

Since severe asthenozoospermia and sperm mitochondrial dysfunction have been clearly associated with oxidative stress and its related structural damage to the sperm (21), we have examined whether sperm alterations in this specific patient was associated with oxidative damage. Neither lipid peroxidation of the sperm membrane, as assessed by TBARS monitoring, nor reactive oxygen species (ROS) were involved. These observations suggest that in the PCD phenotype, spermatozoa is not associated with seminal oxidative stress that could potentially result from defective mitochondrial activity. However, we have observed some damage to the sperm DNA, as reflected in the moderately higher than normal DFI of 33%, currently considered within a pathological range, but unlikely to explain total fertilization failure through IVF/ICSI (22). Since there is no evidence of seminal oxidative stress or lipid peroxidation that could partially explain the increase in sperm DFI, by collecting information from the electron microscopy analysis demonstrating defective sperm nuclear condensation and the presence of nuclear vacuoles, it is possible that the installation of the PCD phenotype during the late phase of spermatogenesis cyto-differentiation (i.e. during spermiogenesis) may result in a particular type of abnormal sperm nuclear packaging which could explain the observed increase in DFI, but this mechanism remains uncertain, and needs longitudinal studies to describe the relationship between PCD and DFI. As high sperm DNA fragmentation is somehow associated with ICSI failure (22, 23), this might help to explain the low to very low successful pregnancy rates when ICSI is performed and suggests DNA evaluation be routinely added to the workout in PCD patients.

### PCD patient management and recommendation

Male patients with severe asthenozoospermia or complete sperm immotility deserve special attention when any of the following factors are present: (i) unexplained neonatal respiratory distress (birth at term), (ii) any lack of organic laterality, such as situs inversus totalis, situs ambiguous or heterotaxis, (iii) year round wet cough, and (iv) year-round nasal congestion (24). Any of these situations should trigger the search for PCD including Kartagener Syndrome. The diagnosis of PCD can then be confirmed either by the search for bi-allelic mutations in known PCD genes, such as DNAH5 (5p15-5p14), DNAH11 (7p21) and DNA11 (9p13-21), representing 15-21%, 6-9% and 2-10% of PCD cases, respectively, or by a classical ciliary ultrastructural defect of PCD observed under a transmission electron microscope (5, 24). Since the disorganization and dysfunction of the mitochondria partly explain the inability of PCD spermatozoa to fertilize, the extent to which this phenotype is present in PCD patients could be systematically studied. The evaluation of the potential of the mitochondrial membrane of sperm by flow cytometry and JC-1 probe (25), the use of mitochondrial-specific probes such as the various commercially available Mitotracker probes, mitochondrial DNA tests or electron microscopy as performed in this study could be useful in assessing the extent of mitochondrial defect in patients with PCD.

In addition, since it is nowadays clear that PCD is not only a problem of sperm motility, other semen parameters and functional characteristics should be evaluated. With regard to our illustrative case study and supposing that the combination of cellular and pathophysiological events made here were to be extended to any patient with PCD phenotype an association with a moderately high level of DNA fragmentation, probably not of oxidative origin, could be a valuable information for the clinician and the couple before any therapeutic and/or corrective action. It is now clear in the community that when a semen sample has a DFI greater than 25-30%, the chances of successful reproduction by ICSI are reduced. DNA damage and, in particular, high DFI have been linked to low fertility rates, impaired embryonic development, pregnancy loss, birth defects and the development of various forms of morbidity in children, including autism and childhood cancer (26-30). Knowing in advance, before any decision is made about the choice of ART therapy or if any at all would be advised, the level of DNA damage and most importantly, the lack of mitochondrial activity in the initial semen samples would have been an important criterion for delivering an appropriate message to the couple to perform electron microscopy in order to gather more useful information. EM results gave fundamental insights as to how important is the lack of proper centriole placement and function in the genesis of fertilization failure or absence of embryo development. This mandatory physiological step most likely could be one of the reasons why this case could not progress to normal embryo development, as illlustrated by the clear centriolar defects and dysfunction that lead to inability to form the mitotic spindle responsible for the opposition and alignment of both male and female pronuclei that will ultimately fuse as a new embryonal genome, or even if they were indeed formed, in the lack of progress in in early stages of embryonal development. Finally, the fibrous sheath dysplasia was the most visible and evident ultrastructural morphological defect and is a key element in the lack of reproductive success, not always found in similar cases. One cannot stress the importance of proper andrological investigation, if not for all that we have demonstrated above, simply for limiting the frustration, anxiety and costs associated with unsuccessful assisted reproductive therapies to individuals and/or society. In this particular case, the only advice given to the couple was to continue IVF/ICSI attempts until they were successful, after a single and simple basic semen analysis, not even the diagnosis of PCD was raised by the attending reproductive gynecologist. The consequence of such inadequate treatment was a divorce for the couple in question mainly because of their inability to cope with this blind stressful situation.

## CONCLUSIONS

Even though the real success rates of 25-30% for all ARTs, including ICSI, still are the same and remains almost unchanged in the last decade, regardless of adding more expensive equipment and complementary techniques, they maintain attractiveness to some IVF clinics because they do not need to invest extra time, resources and knowledge to offer a myriad of other better cost-effective treatments that would be shared among other specialists, including the andrologist and the general gynecologist. For the desperate couple, the illusive promise of a silver-bullet is attractive. However, irrespective of the acknowledged merit, in special when dealing with severe to extreme cases, the “precautionary principle” must be seriously applied and exercised in how ARTs are used and abused. The situation described here, illustrates the type of ultrastructural defects in the male gamete unlikely to be bypassed even employing the most advanced technologies and tools available. Moreover, although the birth of an ART-child can be considered an end-point success, full happiness can only be achieved later, when considering the child's development, life expectancy without major diseases and disabilities, and his or her own ability to be a father or mother, preferably naturally. All these events are controlled both by the child's environment through epigenetics and by his own genetics, the latter being the result of the fusion of the paternal and maternal chromosome moieties at the time of fertilization. If the paternal chromosomal lot is compromised, as appears to be the case in this example of PCD with dysplasia of the sperm fibrous sheath, and if the capacity of oocytes to repair is not optimal, fertilization will eventually result in an embryo carrying defective genetic/epigenetic information that will be transmitted to subsequent generations. Modern andrology, its clinicians and field scientists have the power and the tools to limit these complications by adopting a more rigorous assessment, especially that of the male gamete. In particular, an assessment of the sperm functional aspects, the presence of ROS, LPO and integrity of the nucleus/DNA; not limited to severe infertility, whether due to asthenozoospermia, severe oligozoospermia, teratozoospermia, leukocytospermia or any chronic disease. The final question is: with all the investigative armamentarium available today in modern andrology, can the lack of proper investigation of the male patient and of the male gamete, like presented here, and proceeding directly to unrestricted use of IVF/ICSI be classified as malpractice conduct and the hidden consequences to the offspring in the future be retrospectively implicited to the attending professionals due to the existance of unemployed means, methods and tests to avoid serious consequences? It is passed time to change.
